# Correlation between velocity encoded cine magnetic resonance imaging and Doppler echocardiography for the evaluation of diastolic dysfunction

**DOI:** 10.1186/1532-429X-11-S1-P37

**Published:** 2009-01-28

**Authors:** Ijaz Ahmad, Mitra Sahebazamai, Lesan Banko, Joshua A Sockolow, Igor Klem, Terrence Sacchi, John F Heitner

**Affiliations:** 1 New York Methodist Hospital, Brooklyn, NY USA; 2 Duke University Medical Center, Durham, NC USA

**Keywords:** Leave Ventricular Ejection Fraction, Pulmonary Vein, Cardiac Magnetic Resonance, Ventricular Ejection, Diastolic Dysfunction

## Background

Doppler echocardiographic (Echo) measurements of the mitral and pulmonary venous flow have been used to assess diastolic dysfunction (DD). Velocity-encoded cardiac magnetic resonance (Ve-CMR) can measure similar parameters and may potentially be used for the assessment of DD.

## Objective

To assess the correlation of Ve-CMR with Echo in assessing mitral inflow E/A ratio, left atrial (LA) size, and pulmonary vein (PV) flow.

## Methods

We prospectively enrolled 22 outpatients with a mitral inflow pattern by Echo that was normal (8), impaired relaxation (7), pseudo-normal (2), or restrictive (5). The mean left ventricular ejection fraction (LVEF) was 60.8 and two patients had left ventricular hypertrophy on echo. Ve-CMR was performed within 2 hours of the Echo. The following indices were measured: LA size, mitral inflow E-velocity (E), A-velocity (A), E/A ratio, PV waves (S, D and A).

## Results

**Figure 1:**
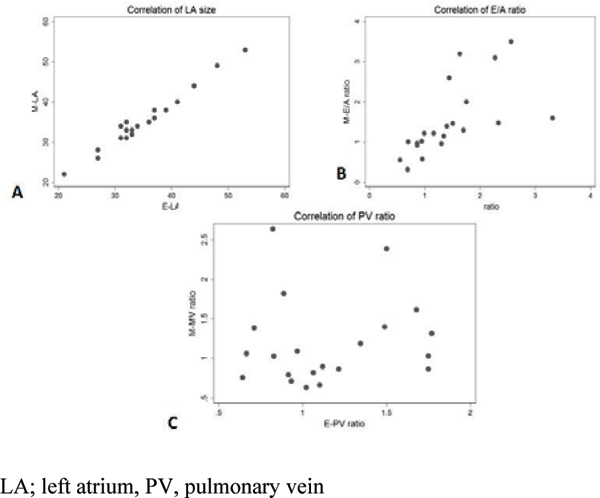
**Correlation between Ve-CMR and Doppler echo derived values was tested using Spearman's rank correlation coefficients**. There was significant correlation between left atrial size and E/A ratio. **A**: LA size (R = 0.9, p-value < 0.05), **B**: E/A ratio (R = 0.88, p-value < 0.05), but not **C**: (R = 0.12, p = 0.58). LA; left atrium, PV, pulmonary vein.

The mean age of the patients was 51 years. The average Echo mitral inflow absolute velocities were close to double the Ve-CMR {Echo E-velocity = 85.8 cm/sec vs. Ve-CMR E velocity = 41.4 cm/sec, Echo A-velocity 70 cm/sec vs. Ve-CMR A-velocity 32.5 cm/sec}. The average E/A ratio was 1.41 by echocardiogram and 1.48 by Ve-CMR. There was a significant correlation in LA size and Mitral inflow E/A ratio between Ve-CMR and Echo (Rho = 0.9, p < 0.01; Rho = 0.88, p < 0.01, respectively). There were no significant correlations in PV waves (Rho = 0.12, p = 0.58). Figure 1

## Conclusion

Ve-CMR has a very high correlation with Echo in the assessment of mitral inflow E/A ratio and LA size. Ve-CMR maybe useful for the assessment of diastolic dysfunction.

